# Plastic bronchitis in an adult

**DOI:** 10.1002/rcr2.70063

**Published:** 2024-11-18

**Authors:** Harshana Bandara, Michael D. Davis, Stephen J. Fowler

**Affiliations:** ^1^ Northwest Lung Centre, Wythenshawe Hospital Manchester University NHS Foundation Trust Manchester UK; ^2^ Hermans B Wells Center for Paediatric Research Riley Hospital for Children at Indiana University School of Medicine Indianapolis Indiana USA; ^3^ Division of Infection, Immunity and Respiratory Medicine, School of Biological Sciences The University of Manchester Manchester UK

**Keywords:** airway obstruction, bronchial casts, lymphoma, lymphoproliferative, plastic bronchitis

## Abstract

Plastic bronchitis is rare in adult pulmonology and has a wide range of aetiology. Cast analysis is key in narrowing down the differential diagnosis of plastic bronchitis. If suspected of having lymphocytic PB, complete imaging to evaluate thoracic lymphatics is important to find out the potential causes for PB.

## Graphical abstract

We present a patient who had expectorated a bronchial cast (plastic bronchitis). The rarity of this entity in adult pulmonology and highlighting the importance of cast analysis and the thoracic lymphatic imaging is brought to the attention of clinicians.

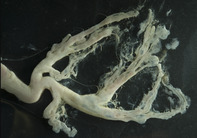

## CLINICAL IMAGE

**FIGURE 1:**
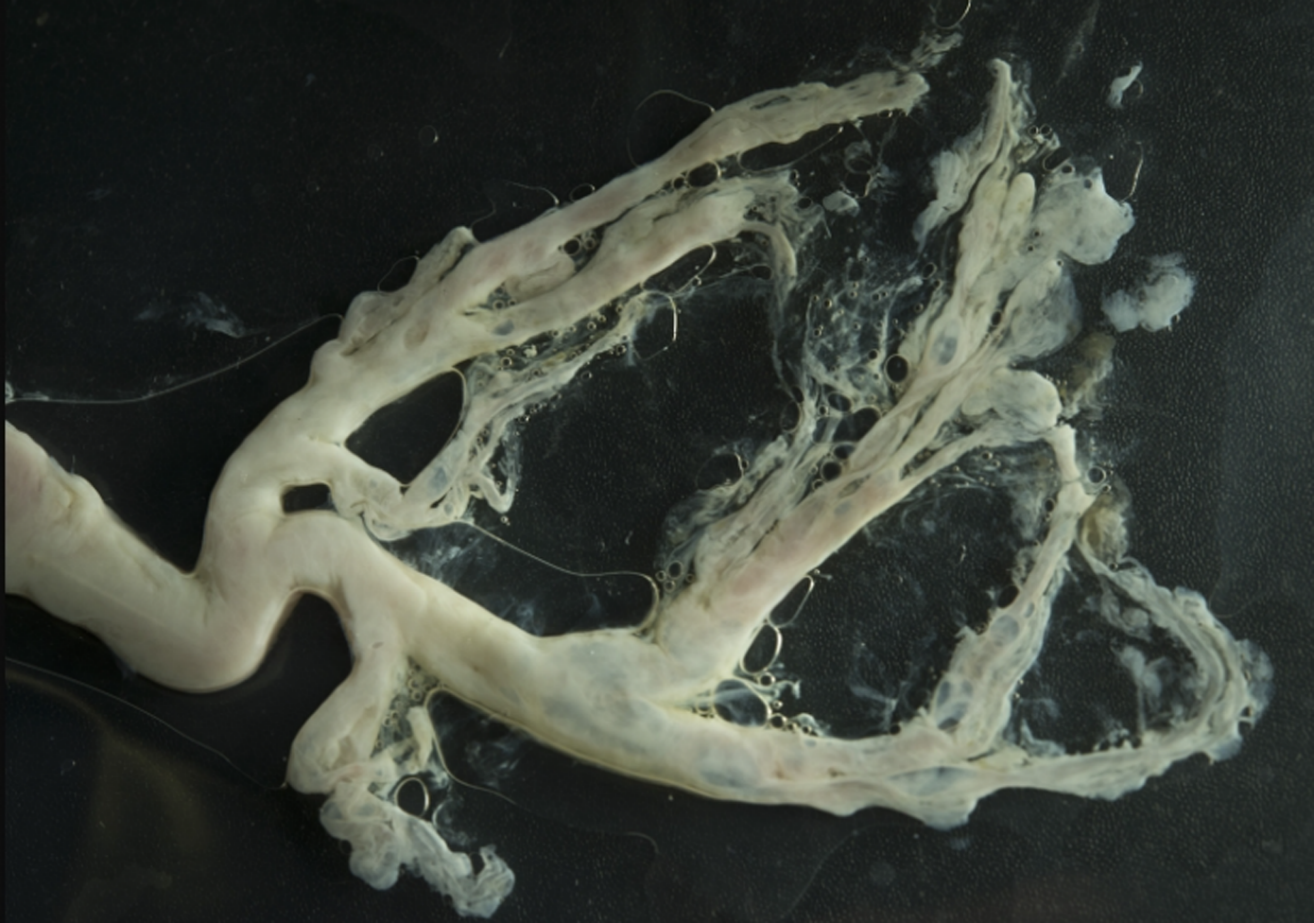
The expectorated cast resembling bronchial anatomy.

**FIGURE 2:**
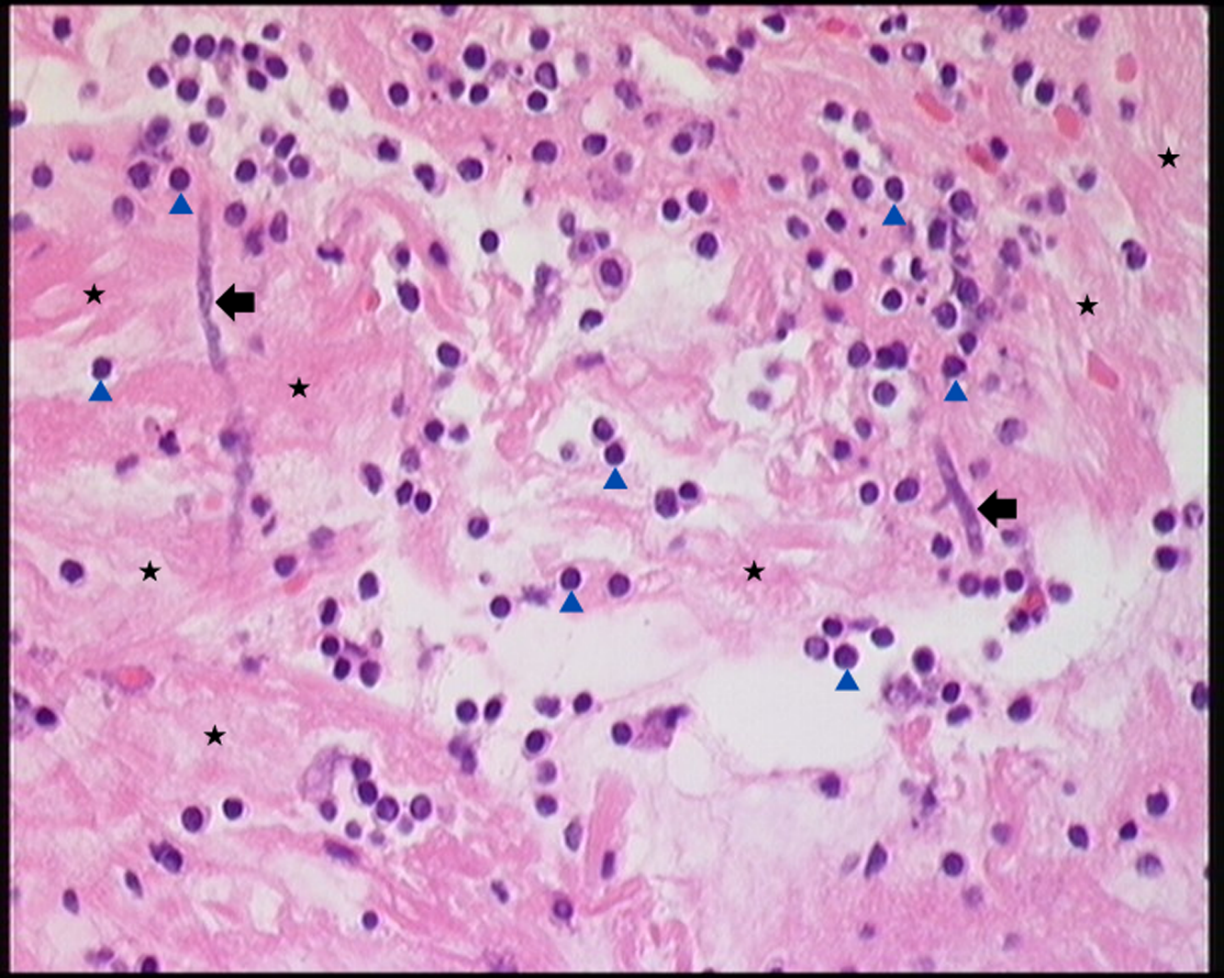
Microscopy (×400) of the expectorated cast showing fibrinous material entrapped (black star) predominantly with lymphocytes (blue arrowhead), suggesting plastic bronchitis. Degraded fungal hyphae (black arrow) are also visible.

A 72‐year‐old male had been treated for eosinophilic bronchitis (4% sputum eosinophils, normal spirometry) with predominant mucous production. He continued to expectorate thick, sticky mucous including casts resembling bronchial anatomy (Figure 1). A few days before the event, he had sub‐acute worsening of respiratory symptoms which partially subsided following the expectoration of the bronchial cast. His vital parameters remained stable. He was treated with a short course of oral corticosteroids to control suspected underlying airways disease, along with a course of empirical oral antibiotics and taught chest clearance manoeuvres. He did not require bronchoscopic removal of casts. Computed‐tomographic imaging showed stable mediastinal and right hilar lymphadenopathy. The histological cast analysis showed abundant fibrinous material with predominant lymphocytes and some interspersed neutrophils, macrophages, and occasional eosinophils. The CD3 and CD20 staining indicated a mix of T/B lymphoid cells. There were no Charcot–Leyden crystals, and the overall histology supported a diagnosis of plastic‐bronchitis (Figure 2). Therefore, based on the clinical evidence and histology the diagnosis of plastic‐bronchitis was made, and he did not cough out casts again while continuing the medical management as same. Specific lymphatic imaging was not available. On follow up, he remained clinically stable, as did the mediastinal adenopathy. Regular inhaled corticosteroids for his airway disease were continued along with carbocisteine and chest clearance techniques. He remained stable despite ongoing milder respiratory symptoms. Though variable mucous burden continued he never again coughed out large bronchial casts.

Seven years later, he developed extra‐thoracic lymphadenopathy, and an inguinal lymph node biopsy and immunoglobulin profile confirmed the diagnosis of Waldenstrom‐Macroglobulinaemia. likely unrelated and incidental. However, this highlights the occurrence of PB in adults and importance of cast analysis, and consideration of lymphatic imaging where available. His care continues under a haematologist, taking an observational approach for the Waldenstrom‐Macroglobulinaemia. His respiratory symptoms have remained stable and controlled.

Plastic bronchitis (PB) is characterized by production and expectoration of branching gelatinous mucous casts which resemble the shape of the airway.^1^, ^2^ The most common occurrence of PB is in children following corrective cardiac surgery.^1^ Adult PB is extremely rare, can be life threatening (mortality as high as 60%^2^), and heterogenous in aetiology. Classification systems vary, but broad categories of “lymphatic” or “non‐lymphatic” are commonly described.^1^, ^2^ Aetiologies in cast formation in adults are categorized in Table 1.

**TABLE 1:** Aetiologies of respiratory cast formation in adults (populated by the submitting authors based on References 1, 2 and 3).

Categories	Disease associations
Anatomical lymphatic obstructions	Primary
	Thoracic duct stenosis
	Thoracic duct duplication
	Secondary
	Post‐surgical
	Coronary artery bypass graft
	Bilateral lung transplantation
	Fontane procedure: Adult onset
	Post tracheoesophageal fistula repair
	Lymphangiectasia
	Lymphangiomatosis
Infection‐related	Viral
	Influenza and HIV
	Bacterial
	*Streptococcus* Spp. and *Haemophilus* Spp.,
	Mycobacterial infections
Haematological	Sickle cell acute chest syndrome
Malignancy‐related	Kaposi sarcoma
	Endobronchial renal cell metastasis
Occupational	Silicosis
Eosinophilic‐diseases	Asthma
	Eosinophilic pneumonia
	Allergy broncho pulmonary aspergillosis
Miscellaneous	Post mantle field radiation for lymphoma
Idiopathic	

*Note*: Aetiologies in cast formation in adults are categorized in the table.

## AUTHOR CONTRIBUTIONS

Harshana Bandara drafted the manuscript. Michael D. Davis and Stephen J. Fowler revised the manuscript. Stephen J. Fowler involved in funds requisition. All authors approved the final manuscript.

## CONFLICT OF INTEREST STATEMENT

None declared.

## ETHICS STATEMENT

The authors declare that appropriate written informed consent was obtained for the publication of this manuscript and accompanying images.

## Data Availability

Data sharing not applicable to this article as no datasets were generated or analysed during the current study.
